# Supplementary material for: Safety and Osteointegration of Titanium Screws Coated with a Fibroblast Growth Factor-2–Calcium Phosphate Composite Layer in Non-Human Primates: A Pilot Study
Source: J Funct Biomater. 2023 May 8;14(5):261. doi: 10.3390/jfb14050261 (PMC10219525; doi:10.3390/jfb14050261)
Supplement: Supplementary file 1 [file jfb-14-00261-s001.zip › jfb-2213033-supplementary.pdf]

Journal of *Functional Biomaterials*

Article

Supplementary Materials: Safety and Osteointegration of Titanium Screws Coated with a Fibroblast Growth Factor-2–Calcium Phosphate Composite Layer in Non-Human Primates: A Pilot Study

Yukei Matsumoto [1], Hirotaka Mutsuzaki [2,3,*], Yuki Hara [1], Katsuya Nagashima [1], Eriko Okano [1], Yohei Yanagisawa [1], Hiroshi Noguchi [1], Tadashi Sankai [4] and Masashi Yamazaki [1]

The Bone apposition rate Weibull plot for control (Figure 6) could be regressed by a bent line with slopes (m) of m > 1 and m < 1 as shown in Figure S1, which is indicative of the mixed or multi-modal Weibull distribution. Thus, the bent line is indicative of involvement of different mechanisms of causing impaired bone apposition in between the lower σ (lnσ < −2.34) and the higher σ (lnσ > −2.34) regions.

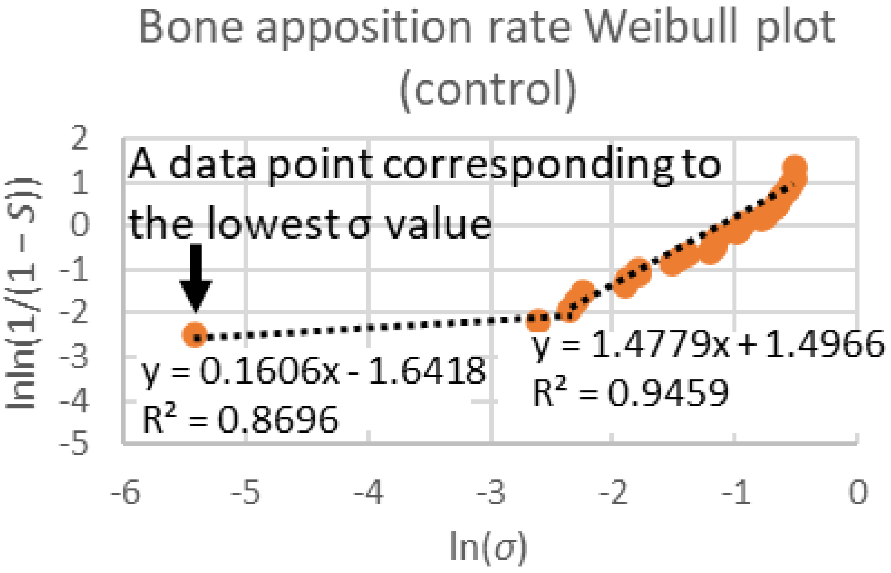

Figure S1. Bone apposition rate Weibull plot for control fitted with a bent line. A inflection point is set at lnσ = −2.34.

However, it is unable to rule out that the bent line is an artifact. For bone apposition rate Weibull plot in the control group, we have 34 data of σ, among which two σ data are zero meaning that three physicians agreed that they are zero. These two data of σ = 0 were excluded from Weibull plot, as lnσ is invalid. However, in one of 34 σ data, two physicians judged as σ= 0 whereas one physician judged as σ = non-zero (0.013158), which gives an extremely low σ value (σ = 0.004385: lnσ = −5.4) after averaging over the three physicians. The averaged value gives a data point corresponding to the lowest σ value in the bone apposition rate Weibull plot (Figure S1).

The data point corresponding to the lowest σ value critically determines the slope m of the regression line. If the judgement of σ = 0 made by the two physicians is true and that made by the third physician is false, the true σ value is zero instead of 0.004385. Then the data point is excluded from the Weibull plot since lnσ is invalid, giving a straight line with a slope of m > 1 as shown in Figure S2 left. If the judgement of σ = 0 made by the two physicians is false and that made by the third physician is true, one can regard σ as

J. Funct. Biomater. **2023**, *14*, 261. https://doi.org/10.3390/jfb14050261

0.013158 (without averaging) instead of 0.004385. In this case, the data points almost fall in a straight line again with a slope of m > 1 (Figure S2 right).

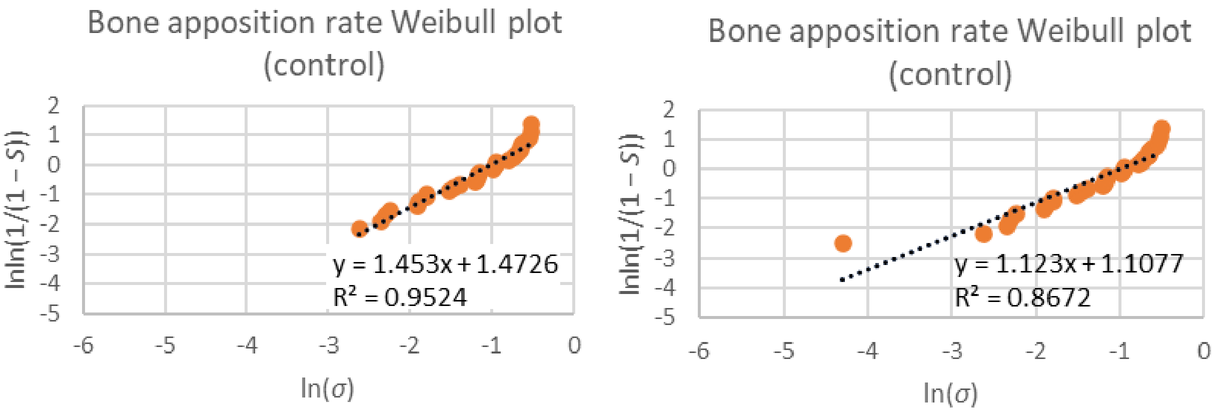

Figure S2. left : Bone apposition rate Weibull plot for control based on the assumption that the judgement of σ = 0 made by the two physician is true and that made by the third physician is false on the data point corresponding to the lowest σ value. Right: Bone apposition rate Weibull plot for control based on the assumption that the judgement of σ = 0 made by the two physician is false and that made by the third physician is true on the data point corresponding to the lowest σ value.

Therefore, on the one hand, bone apposition rate Weibull plots for control could show qualitatively different modes of causing impaired bone apposition in between the lower σ and the higher σ regions which have the slopes of m < 1 and m > 1, respectively. On the other hand, the slope of m < 1 in the lower σ region in bone apposition rate Weibull plots for control could be an artifact resulting from disagreement of judgement on whether σ is zero or non-zero.

The situation is similar to that of the bone apposition rate Weibull plot for the FGF-CP group (Figure S3). The Weibull plot could be regressed by a bent line with slopes of m > 1 and m < 1 as shown in Figure S3 upper, which is indicative of involvement of different mechanisms of causing impaired bone apposition in between the lower σ (lnσ<-1.67) and the higher σ (lnσ > −1.67) regions. However, on the data point corresponding to lowest σ value (σ = 0.008393: lnσ = −4.8), two physicians judged as σ = 0 whereas one physician judged as σ = non-zero (0.025879). If the judgement of σ = 0 made by the two physicians is true and that made by the third physician is false, the true σ value is zero instead of 0.008393. Then the Weibull plot gives a straight line with a slope of m > 1 (Figure S3 lower left). If the judgement of σ = 0 made by the two physicians is false and that made by the third physician is true, one can regard σ as 0.025879 (without averaging) instead of 0.008393. In this case, the data points almost fall in a straight line again with a slope of m > 1 (Figure S3 lower right).

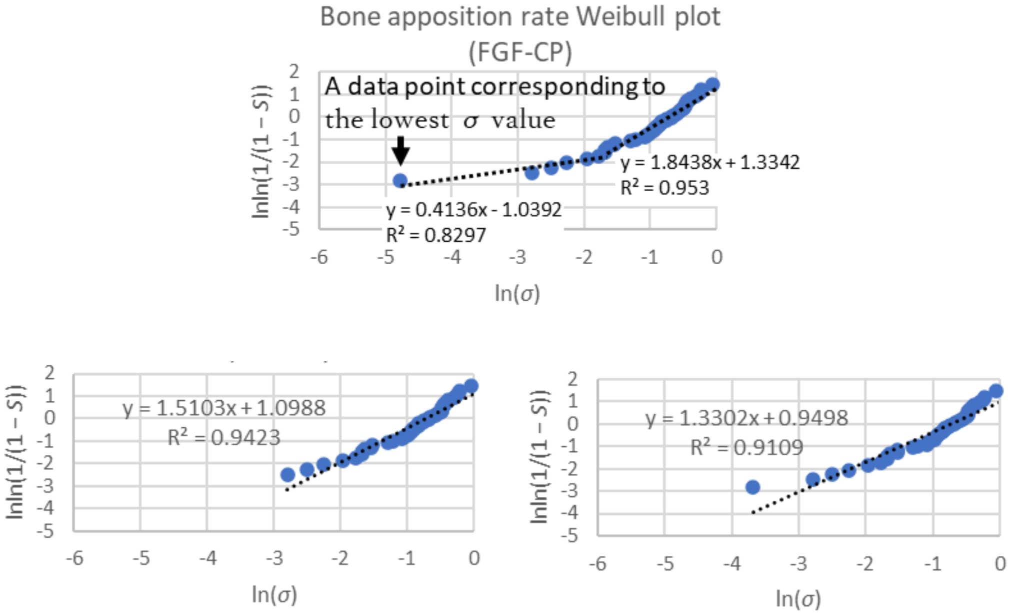

Figure S3. Bone apposition rate Weibull plots for FGF-CP. Upper: a plot fitted with a bent line with an inflection point set at $\ln\sigma = -1.67$. Lower left: a plot based on the assumption that the judgement of $\sigma = 0$ made by the two physician is true and that made by the third physician is false on the data point corresponding to the lowest σ value. Lower right: a plot based on the assumption that the judgement of $\sigma = 0$ made by the two physician is false and that made by the third physician is true on the data point corresponding to the lowest σ value.
